# Deep Sequencing Identifies Tissue-Specific MicroRNAs and Their Target Genes Involving in the Biosynthesis of Tanshinones in *Salvia miltiorrhiza*

**DOI:** 10.1371/journal.pone.0111679

**Published:** 2014-11-03

**Authors:** Xiangbin Xu, Qinghua Jiang, Xiuyan Ma, Qicai Ying, Bo Shen, Yongsheng Qian, Hongmiao Song, Huizhong Wang

**Affiliations:** College of Life and Environmental Sciences, Hangzhou Normal University, Hangzhou, China; Universidade Federal do Rio Grande do Sul, Brazil

## Abstract

*Salvia miltiorrhiza* is one of the most popular traditional medicinal herbs in Asian nations. Its dried root contains a number of tanshinones, protocatechuic aldehyde, salvianolic acid B and rosmarinic, and is used for the treatment of various diseases. The finding of microRNAs (miRNAs) and their target genes will help understand their biological role on the biosynthesis of tanshinones in *S. miltiorrhiza*. In the present study, a total of 452 known miRNAs corresponding to 589 precursor miRNAs (pre-miRNAs), and 40 novel miRNAs corresponding to 24 pre-miRNAs were identified in different tissues of *S. miltiorrhiza* by high-throughput sequencing, respectively. Among them, 62 miRNAs express only in root, 95 miRNAs express only in stem, 19 miRNAs express only in leaf, and 71 miRNAs express only in flower, respectively. By the degradome analysis, 69 targets potentially cleaved by 25 miRNAs were identified. Among them, acetyl-CoA C-acetyltransferase was cleaved by miR5072, and involved in the biosynthesis of tanshinones. This study provided valuable information for understanding the tissue-specific expression patterns of miRNAs in *S. miltiorrhiza*, and offered a foundation for future studies of the miRNA-mediated biosynthesis of tanshinones.

## Introduction

*Salvia miltiorrhiza* Bunge, known as dan shen or tan shen, is one of the important traditional Chinese medicinal herbs. Its dried root is used for the treatment of various cardiovascular diseases, menstrual disorders, blood circulation disturbances, inflammation, apoplexy, and tumor growth [1], [2]. More than 70 components have been isolated and structurally identified from *S. miltiorrhiza* [3]. The main constituents are a number of tanshinones, such as the tanshinone I, tanshinone IIA and tanshinone IIB, and hydrophilic phenolics like the protocatechuic aldehyde, salvianolic acid B and rosmarinic. Tanshinone IIA has effective antioxidant activities, which can inhibit the association of lipid peroxidation products with DNA in liver cells [4], and is widely used to treat various cardiovascular and cerebrovascular diseases [5]. Salvianolic acid B possesses significant antioxidant activities [6], can decrease malondialdehyde level and attenuate brain and heart damages in mice and rabbits [7], [8]. Due to the medicinal importance, *S. miltiorrhiza* becomes one of the most popular traditional herbal medicines in Asian nations, and also is widely accepted as a health supplement in western countries [3], [9]. It has estimated that 20 million kilograms of *S. miltiorrhiza* was needed in China each year. With the growth of annual demand, the amount is still increasing. Hence, enhance yield of *S. miltiorrhiza* or improve content of active constituents in *S. miltiorrhiza* becomes one of the crucial work.

Recent years, small RNAs (sRNAs) are getting more and more attention for their key roles in post-transcriptional or translational gene regulation [10]–[14]. Small interfering RNAs (siRNAs) and microRNAs (miRNAs) are two major groups of sRNAs. MiRNAs are short, single strand and endogenous sRNAs that negatively regulate gene expressions at post-transcriptional level by repressing gene translation or degrading target mRNAs [12], [15]. They are encoded by independent transcriptional units in intergenic regions and transcribed by RNA polymerase II or III to form primary miRNA (pri-miRNA). The pri-miRNA is processed by ribonuclease III enzymes into a stem-loop miRNA::miRNA* duplex of precursor miRNA (pre-miRNA) [16]. In plants, the stem-loop region of pre-miRNAs is cut by Dicer-like (DCL) endonuclease, forming small double stranded RNA (dsRNA) miRNA: miRNA* [12], [16]. The mature miRNA is incorporated in the RNA-induced silencing complex (RISC) with endonuclease AGO and guide the cleavage or translational repression of the target mRNA by complementary base-pairing [17]. MiRNAs are involved in multiple crucial plants biological processes, such as organ development and plant responses to environmental stresses [18]–[20]. They also participate in regulating plant secondary metabolism. For example, cis- and trans-regulation of miR163 and its target genes confers natural variation of secondary metabolites in two *Arabidopsis* species and their allopolyploids [21].

To totally make clear the function of miRNAs on the diverse biological processes, it is essential to exactly identify their target genes and explore their interactions. The recent development of degradome sequencing provided a new method for validation of the splicing targets on a whole genome scale, which revolutionized the traditional computational target prediction and was successfully employed on identifying miRNA targets in plants [22]–[24].

Up to now, miRBase database (20.0, http://www.mirbase.org/) contains 24, 521 miRNA loci from 206 species and 30, 424 mature miRNA products. However, there is still no report of miRNAs from *S. miltiorrhiza*. Identification of the tissue-specific miRNAs and their target genes will help understand their role in a variety of metabolic pathways. Therefore, the goal of this study is to identify tissue-specific miRNAs and their potential targets in *S. miltiorrhiza*, and provide valuable information for future studies of the miRNA-mediated biosynthesis of tanshinones.

## Results

### Analysis of sRNA library data sets

**Figure 1:**
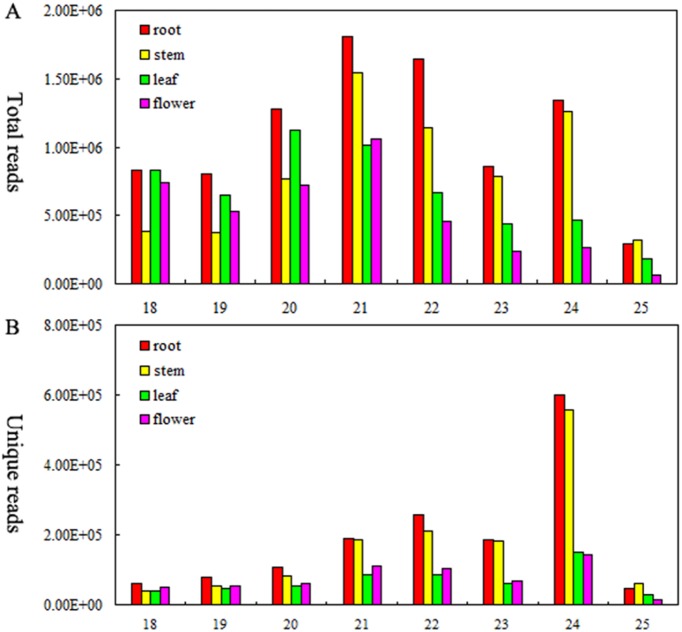
Length distribution of mappable reads of sequ-seqs type in sRNA libraries of root, stem, leaf and flower of *S. miltiorrhiza*. (A) Total mappable reads. (B) Unique mappable reads.

To identify the tissue-specific miRNAs in *S. miltiorrhiza*, four independent sRNA libraries from root, stem, leaf and flower were generated and single-end sequencd (36 bp) on an Illumina Hiseq2500, respectively. The four sRNA libraries yielded a total of 12,385,534, 9,243,928, 9,628,136 and 8,465,920 raw reads, respectively (Table 1). After filtering out the adapter sequences as well as sequences with low quality or low-copy, and further removing mRNA, rRNAs, tRNAs, snoRNAs, snRNAs and other Rfam RNA, a total of 1,524,510, 1,373,067, 553,741 and 601,590 clean mappable sRNA sequences were obtained, respectively (Table 1). The cloning frequency of different sized sRNAs (15–32 nt) was similar among the four libraries. The majority of the sRNAs were 20–24 nt in size (Fig. 1A and B).

**Table 1:** Distribution of the sequences in the four sRNA libraries of root, stem, leaf and flower of *S. miltiorrhiza*, respectively.

Category	Root		Stem		Leaf		Flower	
	Total sequence	Uniq sequence	Total sequence	Uniq sequence	Total sequence	uniq sequence	Total sequence	Uniq sequence
Raw reads	12385534	2215901	9243928	1900101	9628136	974136	8465920	1152808
3ADT & length filter	1973185	613910	1684254	447800	3499909	376162	3698160	508768
Junk reads	19925	12789	20983	14863	8030	4628	6109	4692
Rfam	1493711	62736	939211	62951	735874	38614	681491	36421
Repeats	46248	2327	16801	1726	7029	1208	14897	1624
rRNA	1196844	39138	749931	42718	593104	26292	541802	22135
tRNA	193742	15556	122634	13101	70869	7930	83348	8959
snoRNA	7546	1445	5497	1406	2943	847	3567	1097
snRNA	5057	1804	5594	1654	4678	843	5401	1387
other Rfam RNA	90522	4793	55555	4072	64280	2702	47373	2843
Clean reads	8859267	1524510	6585952	1373067	5378765	553741	4067914	601590

3ADT & length filter: reads removed due to 3ADT not found and length with <18 nt and >25 nt were removed.

Junk reads: Junk: > = 2N, > = 7A, > = 8C, > = 6G, > = 7 T, > = 10 Dimer, > = 6 Trimer, or > = 5 Tetramer.

Rfam: Collection of many common non-coding RNA families except micro RNA; http://rfam.janelia.org.

Repeats: Prototypic sequences representing repetitive DNA from different eukaryotic species; http://www.girinst.org/repbase.

Notes: There is overlap in mapping of reads with rRNA, tRNA, snRNA, snoRNA and repeats.

### Identification of miRNAs

**Figure 2:**
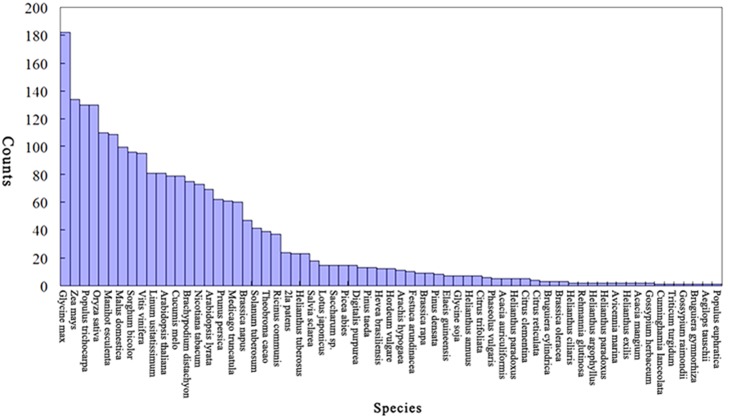
Conservation of the identified known miRNAs in *S. miltiorrhiza*.

To identify the known miRNAs in *S. miltiorrhiza*, the mappable sRNAs were compared with the currently known plant precursor or mature miRNA sequences in miRbase database (20.0, http://www.mirbase.org/). A total of 452 known miRNAs corresponding to 589 pre-miRNAs were identified (Table S1). Among these identified miRNAs, most are highly homologous with that of other plants, such as *Glycine max*, *Zea mays*, *Populus trichocarpa*, *Oryza sativa*, *Manihot esculenta*, *Malus domestica* and *Sorghum bicolor*. In particular, 182 identified miRNAs in *S. miltiorrhiza* are homologous with that of *G. max* (Fig. 2). Base on the sequence similarity, these identified miRNAs could be grouped into 62 miRNA families. Some of the miRNA families, such as miR156, miR159, miR160, miR166, miR169, miR171, miR172, miR319 and miR396, were conserved in 43, 34, 35, 40, 31, 26, 31, 31 and 37 kinds of plant species, respectively (Table S2). Most of the non-conserved miRNAs (miR403, miR408, miR423, miR425, miR436, miR447, miR482, miR528, miR529, miR535, miR812, miR815, miR827, miR845, miR858, miR894, miR951, miR1028, miR1128, miR1509, miR1510, miR1862, miR1863, miR1873, miR1876, miR2111, miR2118, miR2275, miR2673, miR3522, miR3630, miR4243, miR4996, miR5054, miR5059, miR5072, miR5077, miR5139, miR5141, miR5144, miR5145, miR5527, miR5538, miR6173 and miR6478) found in *S. miltiorrhiza* only have been previously identified from a few plant species (Table S2).

Confident annotation of novel species-specific miRNAs requires *dcl1* or *dcl4* knockout mutants [23], [24]. In the absence of these mutants, the ability of the miRNA flanking sequences to fold-back into a stable hairpin structure is an important criterion for the annotation of novel miRNAs. By mapping all sRNA sequences and predicting the secondary structures of the miRNA precursors, 24 pre-miRNAs corresponding to 40 novel miRNAs were first reported in this experiment, of which most were 18, 20 and 21 nt in length (Table S1).

**Figure 3:**
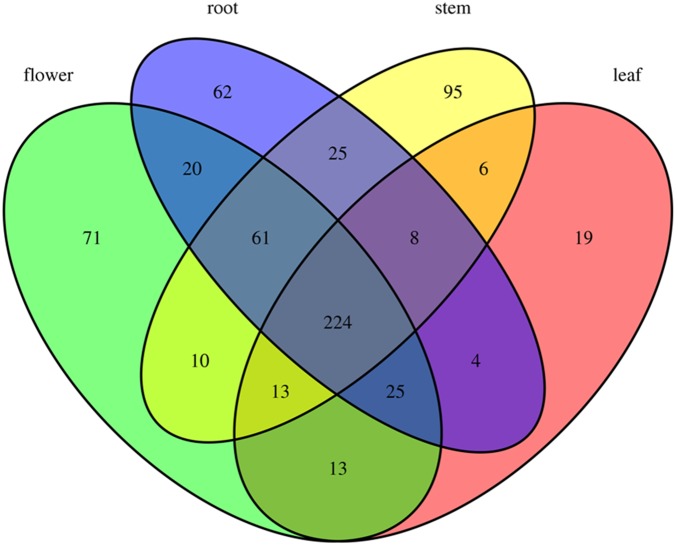
The spatial expression patterns of identified miRNAs in root, stem, leaf and flower of *S. miltiorrhiza*.

Among all the identified known and novel miRNAs, besides 224 miRNAs express in all the tissues, 62 miRNAs express only in root, 95 miRNAs express only in stem, 19 miRNAs express only in leaf, and 71 miRNAs express only in flower (Fig. 3, Table S3), and most of them presenting at a low expression level. Moreover, there are still many miRNAs express in 2 or 3 different tissues, respectively (Fig. 3).

### Differential expression of miRNAs in *S. miltiorrhiza*

**Figure 4:**
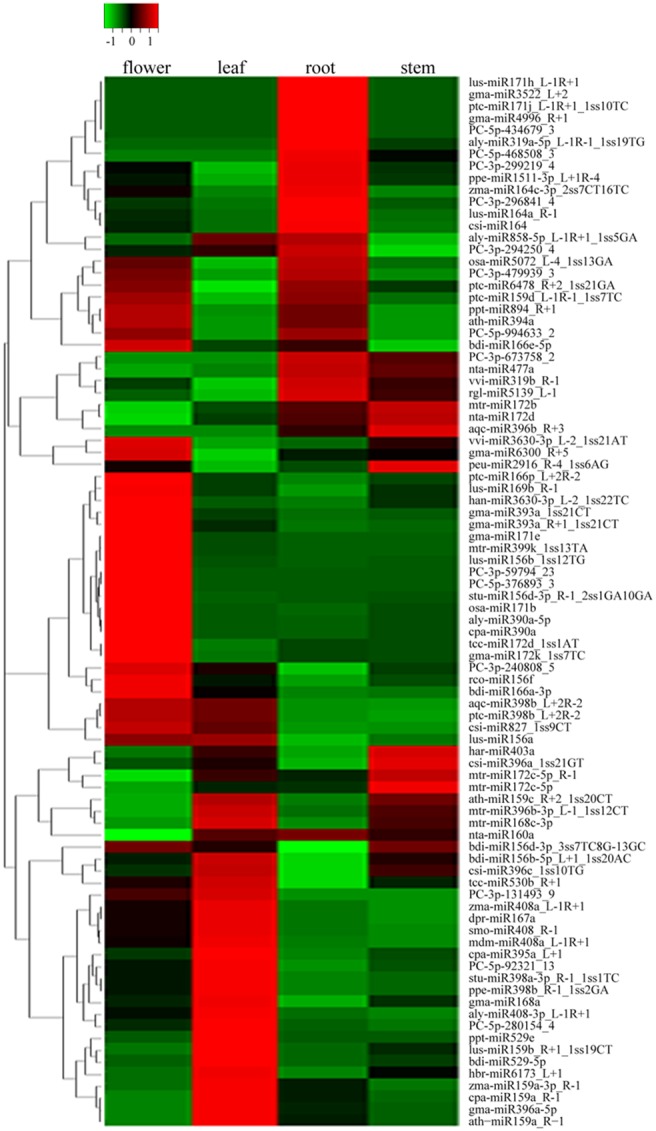
Differential expression levels of miRNAs in root, stem, leaf and flower of *S. miltiorrhiza*. The expression of miRNAs was shown as Z-value. The significance threshold was set to be 0.01. The Z-value of miRNAs higher than 1 was designated as up-regulated, and less than −1 was designated as down-regulated.

To further identify the tissue-specific miRNAs in *S. miltiorrhiza*, the differential expression of miRNAs was analyzed by selectively using Chi-squared test and ANOVA test based on normalized deep-sequencing counts. The significance threshold was set to be 0.01. The Z-value of miRNAs higher than 1 was designated as up-regulated, and less than −1 was designated as down-regulated. As shown in figure 4, 17, 7, 28 and 25 miRNAs were up-regulated in root, stem, leaf, and flower, respectively, and 8, 3, 8 and 5 miRNAs were down-regulated in root, stem, leaf, and flower, respectively.

**Figure 5:**
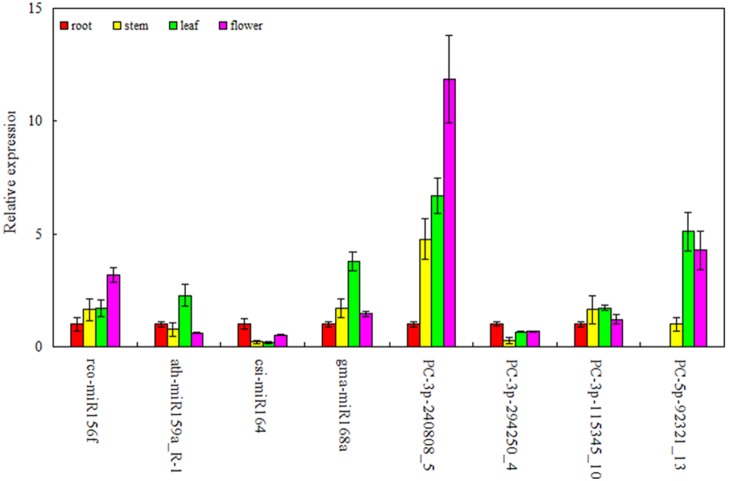
Expression analysis of miRNAs in root, stem, leaf and flower of *S. miltiorrhiza* by RT-qPCR. The amount of expression was normalized by the level of *actin* in RT-qPCR. All reactions of RT-qPCR were repeated three times for each sample.

To validate the sequencing results and the expression level of miRNAs, four known and four novel miRNAs displaying differential expression pattern in different tissues of *S. miltiorrhiza* from the high-throughput sequencing were selected for quantitative real-time PCR (RT-qPCR) analysis. As shown in figure 5, although some of the miRNAs were identified in low reads, such as PC-3p-115345_10 and PC-5p-92321_13, the eight miRNAs RT-qPCR qualitative results was consistent with the data from high-throughput sequencing (Table S1).

### Identification of targets cleaved by miRNAs

To further understand the biological function of these identified miRNAs in *S. miltiorrhiza*, the high-throughput degradome sequencing approach was adopted to perform a genome-wide analysis of the mRNAs potentially cleaved by the miRNAs. In total, 13,381,623 raw reads were obtained from the *S. miltiorrhiza* degradome library (mixture of root, stem, leaf and flower) (data not shown). After removing the reads without the CAGCAG adaptor, 2,287,895 unique raw reads were mapped to the genome database (http://www.ncbi.nlm.nih.gov). The CleaveLand 3.0 was adopted to identify the sliced targets. The cleavage signature for most of the miRNAs was not detectd in this degradome library, only a total of 69 targets potentially cleaved by 25 miRNAs were identified (Table S4). Based on the ‘height’ of the degradome peak at each occupied transcript position, these targets were classified into categories 0, 1, 2, 3 and 4 according to our previous criteria [24], respectively. Among these identified targets, there were 6, 0, 37, 12 and 14 fell into categories 0, 1, 2, 3 and 4, respectively (Table S4). Most miRNAs cleaved two or more different transcription targets, and only five miRNAs cleaved one target. Based on the BLASTX analysis, 29 targets were homologous with the genes that have already been found in other plants (Table 2), including S-adenosylmethionine synthetase, peroxidase, myb proto-oncogene protein, etc.

**Table 2:** Identified miRNA targets in *S. miltiorrhiza* by degradome sequencing.

miRNA	gene	Length	Definition
hbr-miR396a_R-3	gi51954849gbCV165709.1CV165709	437	T-complex protein 1 subunit zeta
ppt-miR166m_R-3	gi51955619gbCV166479.1CV166479	500	S-adenosylmethionine synthetase
ppt-miR166m_R-3	gi51958718gbCV169578.1CV169578	490	S-adenosylmethionine synthetase
ppt-miR166m_R-3	gi51958974gbCV169834.1CV169834	525	S-adenosylmethionine synthetase
ppt-miR166m_R-3	gi51959843gbCV170703.1CV170703	517	S-adenosylmethionine synthetase
osa-miR5072_L-4_1ss13GA	gi51958299gbCV169159.1CV169159	498	peroxidase
osa-miR5072_L-4_1ss13GA	gi51958400gbCV169260.1CV169260	693	peroxidase
smo-miR396_R+1_1ss7GA	gi51952605gbCV163465.1CV163465	526	nucleolin
smo-miR396_R+1_1ss7GA	gi51953108gbCV163968.1CV163968	420	nucleolin
aly-miR858-5p_L-1R+1_1ss5GA	gi51952052gbCV162912.1CV162912	620	myb proto-oncogene protein, plant
aly-miR858-5p_L-1R+1_1ss5GA	gi51955967gbCV166827.1CV166827	548	myb proto-oncogene protein, plant
aly-miR858-5p_L-1R+1_1ss5GA	gi51957272gbCV168132.1CV168132	490	myb proto-oncogene protein, plant
aly-miR858-5p_L-1R+1_1ss5GA	gi51958934gbCV169794.1CV169794	544	myb proto-oncogene protein, plant
ppt-miR166m_R-3	gi51953523gbCV164383.1CV164383	482	large subunit ribosomal protein L9e
zma-miR482-5p_R-1_1ss1TC	gi51952323gbCV163183.1CV163183	464	elongation factor EF-1 alpha subunit
zma-miR482-5p_R-1_1ss1TC	gi51956430gbCV167290.1CV167290	716	elongation factor EF-1 alpha subunit
zma-miR482-5p_R-1_1ss1TC	gi51958162gbCV169022.1CV169022	496	elongation factor EF-1 alpha subunit
zma-miR482-5p_R-1_1ss1TC	gi51958761gbCV169621.1CV169621	592	elongation factor EF-1 alpha subunit
zma-miR482-5p_R-1_1ss1TC	gi51959562gbCV170422.1CV170422	618	elongation factor EF-1 alpha subunit
zma-miR482-5p_R-1_1ss1TC	gi51960584gbCV171444.1CV171444	617	elongation factor EF-1 alpha subunit
osa-miR5072_L-4_1ss13GA	gi51952295gbCV163155.1CV163155	635	carboxymethylenebutenolidase
mtr-miR2673a_L-1_1ss21AT	gi51951421gbCV162281.1CV162281	645	calmodulin
mtr-miR2673a_L-1_1ss21AT	gi51956749gbCV167609.1CV167609	482	calmodulin
aly-miR319a-5p_L-1R-1_1ss19TG	gi51957938gbCV168798.1CV168798	486	calmodulin
aly-miR319a-5p_L-1R-1_1ss19TG	gi51958940gbCV169800.1CV169800	539	calmodulin
aly-miR319a-5p_L-1R-1_1ss19TG	gi51959542gbCV170402.1CV170402	561	calmodulin
aly-miR319a-5p_L-1R-1_1ss19TG	gi51960600gbCV171460.1CV171460	557	calmodulin
ppt-miR166m_R-3	gi51956695gbCV167555.1CV167555	488	aquaporin TIP
osa-miR5072_L-4_1ss13GA	gi51953064gbCV163924.1CV163924	389	acetyl-CoA C-acetyltransferase

**Figure 6:**
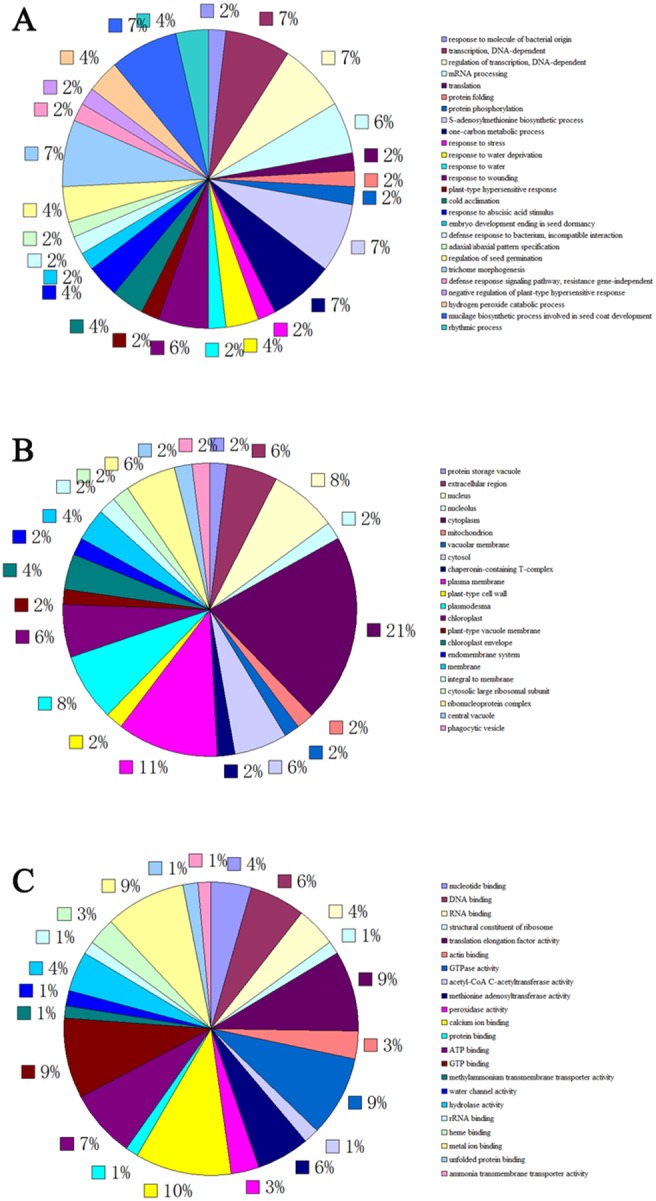
Distribution of the identified targets classified based on putative biological processes (A), cellular component (B), and molecular functions (C).

**Figure 7:**
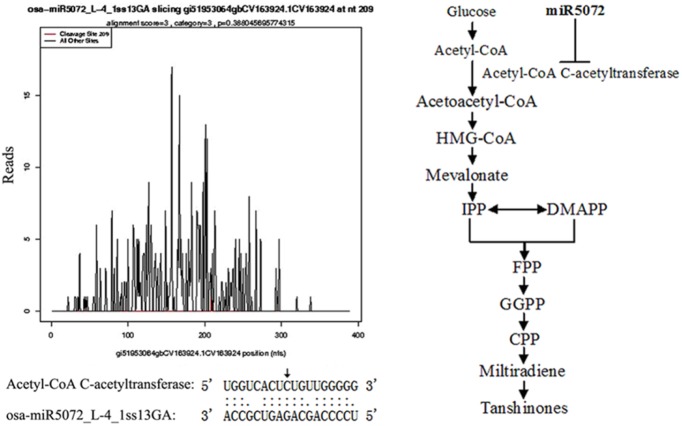
T-plots of *acetyl-CoA C-acetyltransferase* cleaved by osa-miR5072_L-4_1ss13GA and their molecular function in the biosynthesis of tanshinones. (A) T-plots show the distribution of the degradome tag along the full-length of *acetyl-CoA C-acetyltransferase* mRNA sequence (bottom). The red line represents the sliced target transcripts. The alignments show the miRNA with a portion of its target sequence. Two dots indicate matched RNA base pairs; one dot indicates a GU mismatch. The arrow shows the cleavage site. (B) Acetyl-CoA C-acetyltransferase was negatively regulated by miR5072 and involved in the biosynthesis of tanshinones.

To better understand the function of these identified miRNA, their targets obtained from degradome library were subjected to Gene Ontology (GO) analysis and Kyoto encyclopedia of Genes and Genomes (KEGG) Pathway analysis. As shown in figure 6, these targets were involved in 26 biological process, 22 kinds of cellular component, and 22 kinds of molecular function, including binding, peroxidase activity, hydrolase activity, ammonia transmembrane transporter activity, etc. Among these targets, acetyl-CoA C-acetyltransferase (gi51953064gbCV163924.1CV163924), cleaved by osa-miR5072_L-4_1ss13GA, is involved in the biosynthesis of terpenoid backbone and tanshinone (Fig. 7, Table S5).

## Discussion

In the present study, four sRNA libraries from the tissues of root, stem, leaf and flower of *S. miltiorrhiza* were constructed, respectively. A total of 452 known miRNAs corresponding to 589 pre-miRNAs and 40 novel miRNAs corresponding to 24 pre-miRNAs were identified, respectively (Table S1). Most of the identified known miRNA families are highly evolutionarily conserved in a variety of plant species (Table S2), suggesting their conserved roles in plant kingdom. Most of the non-conserved miRNAs, found only in a few plant species, were also identified in *S. miltiorrhiza* (Table S2). It seems likely that these miRNAs relatively recently evolved [25]. They might play important roles in more species-specific characteristics in plant growth and development [26].

Analyzing the temporal and spatial expression patterns of miRNAs would provide useful information about their molecular functions. In plants, more and more evidence showed that miRNAs have differential expression in specific developmental stages and tissues [27]. For example, miR159 mainly expressed in the leaf of potato, and were considered to have crucial function in leaf development [28]. MiR164 mainly expressed in roots of several plant species, and showed essential role in plant root development through their NAC transcription factor targets-mediated downstream pathways [29], [30]. In addition, recent studies also showed that miR166 mainly expressed in barley roots, miR171 mainly expressed in opium poppy roots, miR397 mainly expressed in opium poppy leaves, and miR156 and miR408 mainly expressed in barley leaves [31], [32], which indicated their important roles in plant specific tissues growth and development [26]. In the present study, based on the deep sequencing counts, many miRNAs showed tissue-specific expression in *S. miltiorrhiza.* Among them, 62, 95, 19 and 71 miRNAs only express in root, stem, leaf and flower, respectively (Fig. 3). In addition, miR156 and miR167 were highly abundant in flower and leaf, miR164 were highly abundant in flower and root, and miR166 were highly abundant in all tissues, especially in flower (Table S1), which were quite different from the patterns found in other plants [23], [31]. These suggested that besides some common mechanism sharing with different plant species, there were species-specific miRNA regulatory mechanisms in *S. miltiorrhiza*.

Identification of the targets of these identified miRNAs will help understand their role in a variety of metabolic pathways. By the degradome analysis, 69 targets potentially cleaved by 25 miRNAs were identified, and 29 were homologous with the genes that have already been found in other plants (Table 2). Fortunately, one target of miR5072, acetyl-CoA C-acetyltransferase, was identified in *S. miltiorrhiza*, and related to the biosynthesis of terpenoid compounds and tanshinones in plant (Fig. 7). Tanshinones are abietane-type norditerpenoid quinones identified in root of *S. miltiorrhiza*, which mainly including tanshinone I, tanshinone IIA, dihydrotanshinone I and cryptotanshinone, and showing diverse pharmacological activities, such as antibacterial, antioxidant, antiinflammatory, cytotoxic, neuroprotective, cardioprotective, antiplatelet, and antitumor effects [2], [33]. In plant, diterpenoids are generated from geranylgeranyl diphosphate, which is synthesized from isopentenyl diphosphate and its allylic isomer dimethylallyldiphosphate via two different pathways, the mevalonic acid (MVA) pathway in the cytosol and the 1-deoxy-d-xylulose 5-phosphate (DXP) pathway in cellular plastids [34]–[36]. Generally, the main MVA derived isoprenoid end-products are certain sesquiterpenes, sterols and the side chain of mitochondrial ubiquinones, whereas monoterpenes, certain sesquiterpenes and photosynthesis-related isoprenoids, are derived from the DXP pathway [37]. The two pathways are not separated absolutely. In some extents, there are some forms of crosstalk between them. The isoprenoids generated from MVA can get into the plastid carried by membrane and form monoterpene or diterpene [38]. In the MVA pathway, two units of acetyl-CoA are first catalyzed to acetoacetyl-CoA by acetyl-CoA C-acetyltransferase, and then catalyzed to mevalonate by 3-hydroxy-3-methyl glutaryl coenzyme A reductase (HMGR) [39]. Considering the important role of acetyl-CoA C-acetyltransferase in the initial reaction of MVA pathway, miR5072 must have involved in regulating the biosynthesis of tanshinones in *S. miltiorrhiza*. In addition, acetyl-CoA C-acetyltransferase was also involved in the biosynthesis of a variety of products [40]–[42], indicating the other crucial role of miR5072 in *S. miltiorrhiza*.

Moreover, osa-miR5072_L-4_1ss13GA was also found to target carboxymethylenebutenolidase (Table 2), which known as 4-carboxymethylenebut-2-en-4-olide lactonohydrolase, maleylacetate enol-lactonase, dienelactone hydrolase, and carboxymethylene butenolide hydrolase, belonging to the family of hydrolases. It mainly acts on carboxylic ester bonds and involves in biosynthesis of secondary metabolites. In *S. miltiorrhiza*, it must also have involved in the biosynthesis of active constituents.

In conclusion, for the first time, we obtained 452 known miRNAs and 40 novel miRNAs in *S. miltiorrhiza*, and identified their tissue-specific expression patterns. By degradome analysis, 69 targets potentially cleaved by 25 miRNAs were identified. Among them, acetyl-CoA C-acetyltransferase was cleaved by miR5072 and involved in the biosynthesis of tanshinones. This study provided valuable information for understanding the tissue-specific expression patterns of miRNAs in *S. miltiorrhiza*, and offered a foundation for future studies of the miRNA-mediated biosynthesis of tanshinones.

## Materials and Methods

### Plant material

*S. miltiorrhiza* was cultivated in the experimental field of the Xiasha campus, Hangzhou normal university, China. The root, stem, leaf and flower were collected, immediately frozen and stored at −80°C. No specific permissions were required for these locations/activities, and this study was supported by the open foundation (ZK-14) of the Key Laboratory of Zhejiang Province Medicinal Plants Germplasm Improvement and Quality Control Technology. We confirm that this study did not involve endangered or protected species.

### SRNA library construction and sequencing

Total RNA was extracted using Trizol reagent (Invitrogen, CA, USA) following the manufacturer’s procedure. The total RNA quantity and purity were confirmed with Bioanalyzer 2100 and RNA 6000 Nano LabChip Kit (Agilent, CA, USA) with RIN number >7.0. Approximately 1 ug of total RNA were used to prepare sRNA library according to protocol of TruSeq Small RNA Sample Prep Kits (Illumina, San Diego, USA). And then performed the single-end sequencing (36 bp) on an Illumina Hiseq2500 at the LC-BIO (Hangzhou, China) following the recommended protocol.

### Analysis of sequencing data

The raw reads were subjected to the Illumina pipeline filter (Solexa 0.3), and then the dataset was further processed with an in-house program, ACGT101-miR (LC Sciences, Houston, Texas, USA) to remove adapter dimers, junk, low complexity, common RNA families (rRNA, tRNA, snRNA, snoRNA) and repeats. Subsequently, unique sequences with length in 18–25 nt were mapped to specic species precursors in miRBase 20.0 (http://www.mirbase.org/) by BLAST search to identify known miRNAs and novel 3p- and 5p- derived miRNAs. Length variation at both 3′ and 5′ ends and one mismatch inside of the sequence were allowed in the alignment. The unique sequences mapping to specific species mature miRNAs in hairpin arms were identified as known miRNAs. The unique sequences mapping to the other arm of known specific species precursor hairpin opposite to the annotated mature miRNA-containing arm were considered to be novel 5p- or 3p-derived miRNA candidates. The remaining sequences were mapped to other selected species precursors (with the exclusion of specific species) in miRBase 20.0 by BLAST search, and the mapped pre-miRNAs were further BLASTed against the specific species genomes to determine their genomic locations. The above two we defined as known miRNAs. The unmapped sequences were BLASTed against the specific genomes, and the hairpin RNA structures containing sequences were predicated from the flank 120 nt sequences using RNAfold software (http://rna.tbi.univie.ac.at/cgi-bin/RNAfold.cgi). The criteria for secondary structure prediction were: (1) number of nucleotides in one bulge in stem (< = 12) (2) number of base pairs in the stem region of the predicted hairpin (> = 16) (3) cut off of free energy (kCal/mol < = 15) (4) length of hairpin (up and down stems + terminal loop > = 50) (5) length of hairpin loop (< = 200). (6) number of nucleotides in one bulge in mature region (< = 4) (7) number of biased errors in one bulge in mature region (< = 2) (8) number of biased bulges in mature region (< = 2) (9) number of errors in mature region (< = 4) (10) number of base pairs in the mature region of the predicted hairpin (> = 12) (11) percent of mature in stem (> = 80).

### Verification of miRNAs by RT-qPCR in *S. miltiorrhiza*

To validate the presence and expression of the identified miRNAs, four known and four novel miRNAs were assayed by RT-qPCR, respectively. Total RNA was extracted from the different tissues of *S. miltiorrhiza* with Trizol reagent following the manufacturer’s instructions (Invitrogen, CA, USA). The total RNA (2.5 µg) was treated with DNase I (TaKaRa, Dalian, China) to remove the genomic DNA and reverse-transcribed using miRNA specific primers (Table S6). The forward primers of eight selected miRNAs were designed according to the sequence of miRNA itself, and the universal primer was used as reverse primer (Table S6). 1 µl of cDNA was used as the template in 25 µl PCR reactions which contained 12.5 µl 2×SYBR Premix Ex Taq II (TaKaRa, Dalian, China), 1 µl each of forward and reverse primer (10 µM) and 9.5 µl water. Reactions were performed using the iCycler iQ real-time PCR detection system (Bio-Rad). Each sample contained three replicates of 15 plant tissues. All reactions were performed in triplicate for each sample and *actin7* was used as an internal reference. The threshold value was empirically determined based on the observed linear amplification phase of all primer sets. Sample cycle threshold (Ct) values were standardized for each template based on an *actin7* control reaction. The comparative Ct method (2^−ΔΔCt^) was used to determine the relative transcript abundance of each miRNA [43].

### Degradome library construction, sequencing and target identification

Total RNA was extracted using Trizol reagent (Invitrogen, CA, USA) following the manufacturer’s procedure. The total RNA quantity and purity were confirmed with Bioanalyzer 2100 and RNA 6000 Nano LabChip Kit (Agilent, CA, USA) with RIN number >7.0. Approximately 20 ug of total RNA were used to prepare Degradome library. The method of Ma et al. [44] was followed with some modification. (1) Approximately 150 ng of poly (A) + RNA was used as input RNA and annealing with Biotinylated Random Primers. (2) Strapavidin capture of RNA fragments through Biotinylated Random Primers. (3) 5′ adaptor ligate to only those RNAs containing 5′-monophosphates. (4) Reverse transcription and PCR (5) Libraries were sequenced using the 5′ adapter only, resulting in the sequencing of the first 36 nucleotides of the inserts that represented the 5′ ends of the original RNAs. Then the single-end sequencing (36 bp) on an Illumina Hiseq2500 at the LC-BIO (Hangzhou, China) was performed following the vendor’s recommended protocol. A Public software package, CleaveLand3.0 was used for analyzing sequencing data.

### Accession number

Sequencing data obtained in this work have been submitted to the Gene Expression Omnibus under the accession number GSE60757.

## Supporting Information

Table S1 Profile of known and novel microRNAs identified in *S. miltiorrhiza*.
(XLS)

Table S2 Conservation of the identified known miRNAs in *S. miltiorrhiza*.
(XLS)

Table S3 The tissue-specific miRNAs in *S. miltiorrhiza*.
(XLS)

Table S4 Information of miRNA target genes identified in *S. miltiorrhiza* by degradome sequencing.
(XLS)

Table S5 The KEGG pathway annotation of the target genes identified in *S. miltiorrhiza*.
(XLS)

Table S6 Primers of miRNAs used in this study.
(XLS)
